# 

**Published:** 1895-02

**Authors:** 


					﻿ESTABLISHED 1855.
8UB8CRIPTIOtf, $2-00.
.ATLANTA -
IWEDIGfth AND SURGIGfili
JOURNAL.
OLD SERIES, VOL. XXVI. NEW SERIES, VOL. XI.
LUTHER B. GRANDY, M.D.,
MANAGING EDITOR.
M. B. HUTCHINS, M.D.,
BUSINESS MANAGER.
Atlanta, Ga., February, 1895. No. 12.
				

